# Protein conformational plasticity and complex ligand-binding kinetics explored by atomistic simulations and Markov models

**DOI:** 10.1038/ncomms8653

**Published:** 2015-07-02

**Authors:** Nuria Plattner, Frank Noé

**Affiliations:** 1 Department of Mathematics, Computer Science and Bioinformatics, Free University Berlin, Arnimallee 6, 14195 Berlin, Germany

## Abstract

Understanding the structural mechanisms of protein–ligand binding and their dependence on protein sequence and conformation is of fundamental importance for biomedical research. Here we investigate the interplay of conformational change and ligand-binding kinetics for the serine protease Trypsin and its competitive inhibitor Benzamidine with an extensive set of 150 μs molecular dynamics simulation data, analysed using a Markov state model. Seven metastable conformations with different binding pocket structures are found that interconvert at timescales of tens of microseconds. These conformations differ in their substrate-binding affinities and binding/dissociation rates. For each metastable state, corresponding solved structures of Trypsin mutants or similar serine proteases are contained in the protein data bank. Thus, our wild-type simulations explore a space of conformations that can be individually stabilized by adding ligands or making suitable changes in protein sequence. These findings provide direct evidence of conformational plasticity in receptors.

## Abstract (web summary)

Conformational plasticity influences several aspects of protein function. Here the authors combine extensive MD simulations with Markov state models—using trypsin as model—to reveal new mechanistic details of how conformational plasticity influence ligand-receptors interactions.

## Graphical abstract

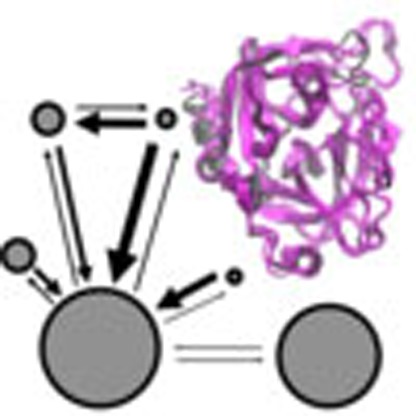

Understanding the process of protein–ligand binding is of fundamental biological interest and essential for structure-based drug design^1^,^2^. It is now realized that not only binding affinities but also kinetics are determining the drug efficacy^3^.

Moreover, the existence of multiple metastable states has been revealed using single-molecule recordings^4^,^5^,^6^,^7^ and highly sensitive NMR experiments^8^,^9^,^10^ in both membrane channels and soluble proteins. A thorough understanding of protein–ligand binding encompasses a complete characterization of the binding-competent conformations of the protein, the binding poses and the complex kinetics between these conformations. Employing this full kinetic network for drug efficacy optimization may significantly enhance our ability to do computational drug design^11^,^12^.

Unfortunately, such a detailed description of the structure–stability–kinetics relationship has as yet been out of reach as no experimental method is available to simultaneously resolve full structures and dynamics, and for molecular dynamics (MD) simulations, it is challenging to escape the long-lived binding states and efficiently sample different binding poses or protein conformations. Extensive sets of MD simulations have been successfully combined with Markov state models (MSMs)^13^ to reveal complex multistate kinetics of folding of peptides and small proteins^14^,^15^,^16^ and conformational changes^17^,^18^,^19^,^20^,^21^,^22^. In ref. 23, distributed computing and Markov models have been used to characterize binding pathways and pre-bound states of the Benzamidine inhibitor to the Trypsin protein. In ref. 24, an extensive data set generated by the Anton supercomputer has been used to study drug binding to a G-protein-coupled receptor. In neither of these studies, however, multiple protein conformations could be identified. In refs 25, 26, the interplay of conformational dynamics and ligand binding in the LAO-binding- and the choline-binding proteins were investigated.

In this paper, extensive MD simulations of ∼150 μs cumulated simulation time of the serine protease Trypsin with its reversible competitive inhibitor Benzamidine are analysed with a Markov model. It is found that Trypsin has multiple long-lived binding-competent conformations. Both open and closed states of binding pocket S1 are found that bind Benzamidine in the crystal structure^27^,^28^. S1 was reported open or semi-open in Trypsin crystal structures; however, closed S1 pockets have been found for Thrombin and other Trypsin-like serine proteases^29^,^30^,^31^,^32^,^33^,^34^,^35^,^36^,^37^,^38^. Thrombin has been characterized to bind according to a conformational selection mechanism and the S1-closed structure has served as a model for the inactive state^31^. In addition to S1-switching, we find a second Benzamidine-binding pocket in Trypsin that is also switchable. At a neighbouring loop, a third conformational switch is found that regulates the binding affinity.

These conformational switches are coupled and give rise to the metastable conformational states found. Although all conformations are found to be binding-competent, their relative stabilities are modulated by the binding of Benzamidine. Moreover, for each such conformation, we can identify one or multiple crystal structures in the protein data bank, including structures of Trypsin, Trypsin mutants or other serine proteases that have equivalent features in their binding pocket structures. This suggests a picture of conformational plasticity. The Trypsin wild-type free energy landscape can be remodelled, thus stabilizing different conformations, by either ligand binding or suitable changes in the protein sequence.

The Trypsin conformations found exchange on very long timescales (tens of microseconds). This gives rise to kinetic partitioning into different binding channels. Our model contains both aspects of conformational selection, a concept where ligands bind to pre-formed receptor conformations^10^,^39^,^40^,^41^,^42^,^43^, and induced fit where the presence of the ligand induces the formation or at least a probability shift of a receptor conformation^42^,^43^,^44^. Different conformations bind the ligand with different affinities, giving rise to a complex multistate picture of binding. This complex kinetic picture has profound consequences for the multiscale description of drug binding to proteins.

## Results

### Markov model

Overall, 543 MD trajectories with a cumulative simulation time of 149.1 μs were generated using an atomistic protein–ligand model with explicit solvent. These data exhibit various events of Trypsin–Benzamidine association, dissociation and conformational changes. A MSM^45^,^46^,^47^,^48^,^49^ was estimated from the simulation data using pyEMMA ( http://pyemma.org). The MSM estimation reweighs the trajectories such that the equilibrium kinetics and distribution among the configurations sampled in the trajectory data can be recovered. After successful statistical validation (Supplementary Figs 3 and 4), the MSM was analysed by computing metastable (long-lived) conformations, their kinetics and equilibrium probabilities, and binding/unbinding pathways (see Methods for details).

**Figure 1:**
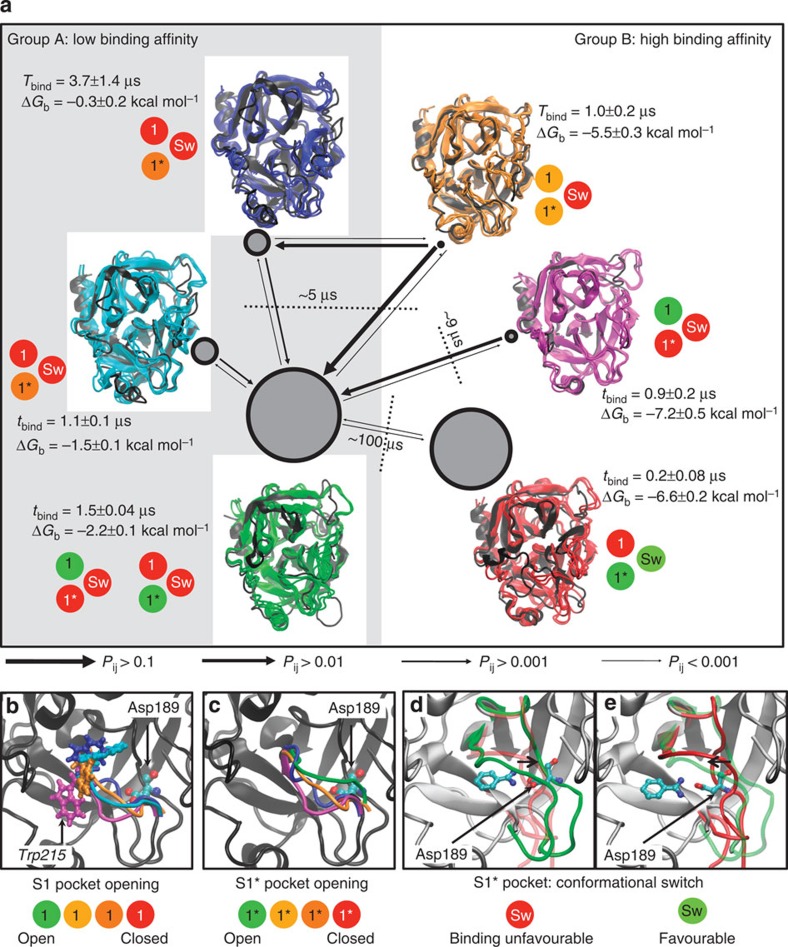
Apo-state structures and kinetics. (**a**) Structural features, equilibrium distribution and kinetics of six unbound (apo) protein conformations. Transitions between them occur at timescales on order of tens of microseconds. The three slowest relaxation timescales and their corresponding transition process are indicated (dashed lines). The circles have an area proportional to the equilibrium probability *π*_*i*_. Their respective free energy differences Δ*G*_b_ of binding a ligand to this conformation and the binding time *t*_bind_ (mean first passage time to binding) are given. The arrows indicate the transition probabilities for direct transitions between the different states (see legend). The most important structural differences concerning ligand binding are shown in **b**–**e**, and the structures are classified with respect to these features in by green/orange/red bullets in **a**. The structures are classified by the state of S1 or S1*: open (green circle with ‘1' or ‘1*'), half-open (orange circle) or closed (red circle) and by the S1* pocket conformational switch: favourable for binding (green circle with ‘Sw') or unfavourable for binding (red circle with ‘Sw').

Configurations were grouped into seven metastable conformations that interchange at timescales of 400 ns or slower. This choice was made as there is a time separation with subsequent relaxation timescales being below 150 ns. This grouping turned out to distinguish different protein conformational states that interconvert slowly, but did not separate bound and unbound states. This is because ligand binding occurs relatively quickly at the simulated system volume. To analyse the binding process, the metastable conformations were further split into bound, unbound and associated (or pre-bound/off-target) sets each. Each microstate was split initially on the basis of the minimal heavy-atom distance between the Benzamidine ligand and the recognition site Asp189 into a bound state and remaining states using a cutoff of 6 Å. The microstates obtained from the MSM were sorted into bound, associated and unbound sets of each metastable state on the basis of the average Benzamindine-Asp189 distance of each microstate being <6 Å, 6–15 Å and>15 Å, respectively. As a result, six apo Trypsin conformations (Fig. 1), seven bound conformations (Fig. 3) and four associated conformations are obtained. Examples for associated states are shown in Supplementary Fig. 5. Most associated states, already described in previous studies^23^, are relatively unstable and short-lived.

### Slowly interconverting apo states

Figure 1 shows the apo state of the metastable Trypsin conformations in different colours. Three representative structures for each conformation are shown, superimposed on the crystal structure 3PTB (in black) for comparison. The conformational changes between different apo conformations are slow—they are governed by relaxation timescales from microseconds to ∼100 microseconds. As the crystal structure 3PTB contains Benzamidine, all of the Trypsin apo conformations deviate from the crystal structure. However, the magenta structure is very similar to 3PTB and only differs by the open conformation of a peripheral loop (top right) that is likely affected by crystal contacts in the periodic cell. This loop change is associated with a different coordination pattern of the calcium ion only observed in the magenta conformation (Supplementary Fig. 6). We consider the magenta structure as ‘X-ray-like'.

Figure 1 also shows the probability of each apo conformation by the area of the disc representing that structure, and the respective binding free energies, that is, the free energy difference for binding to this conformation under standard conditions. The binding affinities vary substantially between different metastable states. The six metastable states can be classified in terms of structural features of two flexible loops, the Trp215 loop and the Asp189 loop (Fig. 1b). The classification is found in Fig. 1a:

1. S1 pocket opening and closing (Fig. 1b and bullets with ‘1' in Fig. 1a): S1 is the Benzamidine-binding pocket found in PDB 3PTB that allows access and hydrogen bonding of Benzamidine to the recognition site Asp189. In the X-ray-like magenta structure the S1-binding pocket is open, whereas in all other structures it is only partially open or closed.
2. S1* pocket opening and closing (Fig. 1c and bullets with ‘1*' in Fig. 1a): a second binding pocket allows binding of Benzamidine to Asp189 from a different angle. In the apo state, an open S1* binding pocket is favourable as seen from the fact that the red and green conformations with this pocket fully open are the two most stable apo states. In the green conformation the pocket can open and close, but microstates with the S1* pocket open have a much higher probability.
3. Conformational switch in the S1* pocket (Fig. 1d,e and bullets with ‘Sw' in Fig. 1a): the binding free energy for binding to S1* crucially depends on its conformation. In the red structure, which is most stable, the loop containing Asp189 is moved inwards making the binding to Asp189 easier, whereas in all other structures it is found at a less favourable position.

In addition, the apo-state conformations differ also in other structural features including the calcium-binding loop (residues 71–79) and the peripheral loop and helix formed by residues 164–177. A direct comparison of these features between the green and the magenta structure is shown in Supplementary Fig. 7.

Interestingly, the X-ray-like apo structure (magenta) is one of the less stable structures in solution. Note that the corresponding X-ray structure includes Benzamidine. Without the ligand, the open state of the S1 pocket is likely unfavourable as it exposes the hydrophobic sidechain of Trp215 to solvent. In contrast, closing the S1 pocket as in the red structure is stabilized by a hydrophobic contacts between Trp215, Val217 and part of Gln192. This finding is in agreement with stabilities of similar conformations in Thrombin as has been revealed by a combination of kinetic measurements and X-ray structures^30^.

The red and green structures differ in the Asp189 loop conformational switch (Sw) and are the two most stable apo structures (together >92% of the apo-state population).

All apo structures are connected via the green conformation, which is the only conformation containing both, structures with an open S1 and a closed S1* pocket as well as structures with an open S1* and a closed S1 pocket. Within the green conformations, these two structures interconvert rapidly. Thus, the green conformation plays a key role here: it is a hub because the ability to form open and closed states in both pockets makes this conformation accessible from all other conformations. Second, it is a transition state that is stable in the apo, but relatively unstable in the bound form, as it exhibits a poor conditional binding affinity. The X-ray-like magenta apo structure quickly relaxes to the more stable green apo structure, but transitioning into the almost equally stable red apo structure is then a slow process, on the order of 100 μs.

The conformational changes observed for the apo states have implications for the function of Trypsin. Trypsin substrates coordinate to Asp189 in the S1 pocket (Supplementary Fig. 1). The opening/closing of the S1 pocket may thus be a mechanism to regulate the protease activity of Trypsin, with effects on the downstream cascade. Conformational changes of the loops forming the S1 pocket may control the selectivity of the protease^50^. Substrate binding can only occur efficiently when Trypsin is in the X-ray-like magenta or the green conformation.

### Observed conformational plasticity versus other sequences

**Figure 2:**
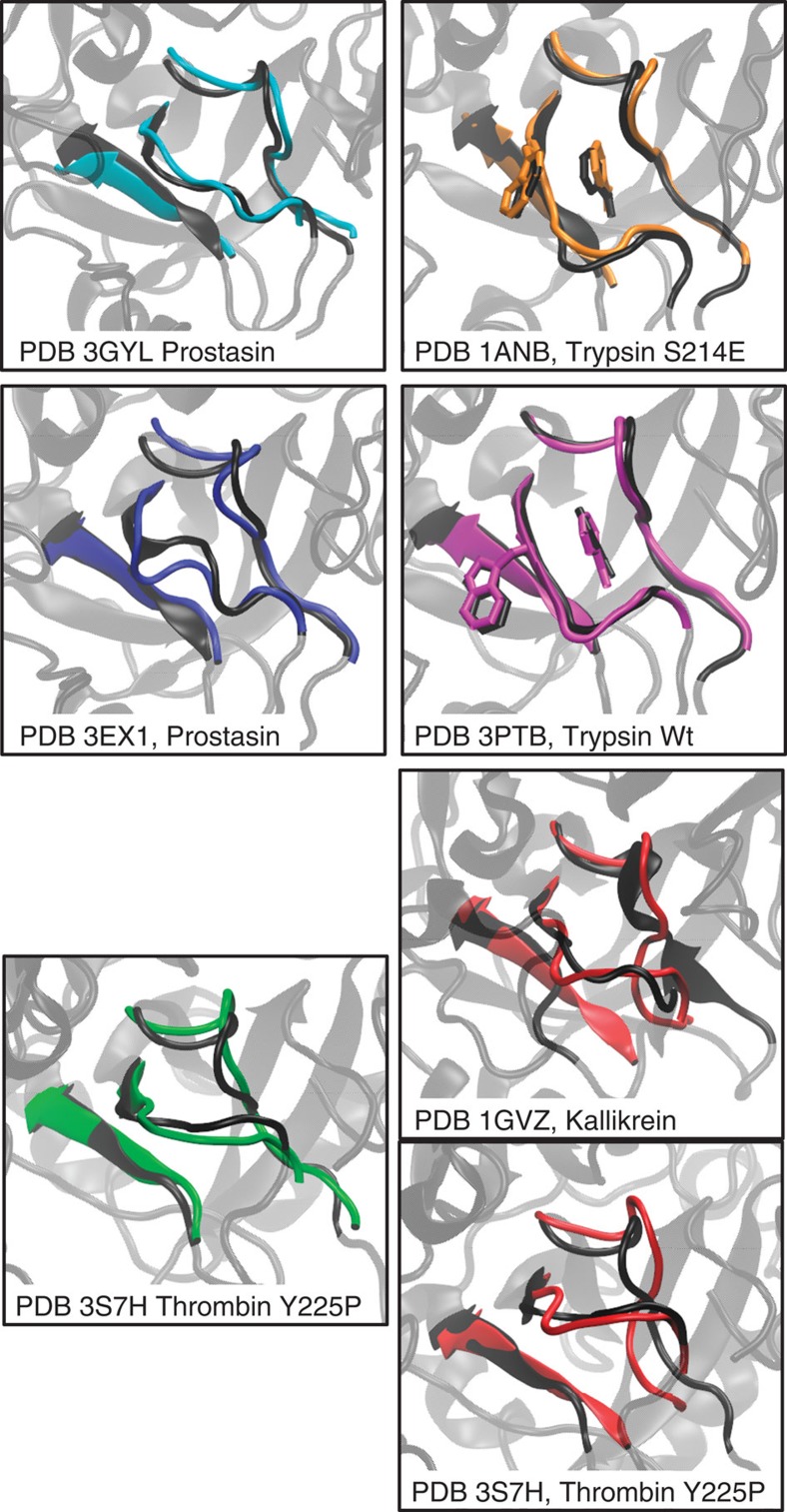
Metastable state conformations compared with serine protease X-ray structures. Conformations found in Trypsin wild type (coloured as in Fig. 1) are matched by crystallographic structures (grey) of other serine proteases. Similar binding site conformations are found in prostasin (blue Trypsin conformations) and the Thrombin Y225P mutant (green) as well as several other Thrombin mutants. The magenta Trypsin conformation corresponds to its wild-type structure in PDB 3PTB. The orange Trypsin wild-type conformation is similar to the X-ray structures of the Trypsin mutants S214E and S214K. The red Trypsin conformations have no equivalent crystallographic structures. Similar to the green state it has similarities to Thrombin Y225P and Kallikrein.

For all of the six metastable apo states in Trypsin, we have found a serine protease X-ray structure with similar structural features at the binding site. The different X-ray structures have been compared with the metastable states by aligning them to PDB 3PTB on the basis of the backbone atoms of the conserved three loops forming the S1 pocket: residues 188–196, 214–220 and 226–230. Figure 2 shows the binding site of these aligned structures together with structures of each metastable state (colours like in Fig. 1) that were aligned to PDB 3PTB in the same way. The Trp215 loop is found in various conformations: in PDB 3PTB (Trypsin wild type) and the magenta Trypsin conformation it is parallel to the Asp189 loop with Trp215 placed outside the S1 pocket. In the orange Trypsin conformation and PDBS 1ANB and 1ANC^28^ (Trypsin mutants S214E and S214K), Trp215 is rotated towards the S1 pocket making the access more difficult and the binding less favourable. In the two blue Trypsin conformations and the prostasin structures 3GYL and 3E1X (ref. 36), the conformation of the Trp215 loops blocks the access to the S1 pocket for Benzamidine and makes the access to the S1* pocket very difficult. In the two prostasin structures, the change in the conformations of the Trp215 loop corresponding to the conformational change between the two blue structures is because of the addition of calcium.

A different conformation of the Trp215 loop closing the S1 pocket is observed in the Thrombin mutant Y225P (PDB 3S7H), several other Thrombin structures^29^,^30^,^31^ and in the green and the red Trypsin conformations. The Asp189 loop conformation of the red Trypsin conformation is not directly found; the most similar conformation is the Kallikrein structure 1GVZ (ref. 33).

Similar conformations of the three loops defining the binding site can be found in the crystallographic structures of other serine proteases, including structure factor D^32^, hepatocyte growth factor activator (HGFA)^34^, *α*I-tryptase^35^ and complement factor I (ref. 37) and various zymogens, for example, pro-granzyme K (ref. 38).

This analysis indicates that Trypsin has a high degree of conformational plasticity: the various conformations accessible to the wild type by thermal fluctuations can be individually stabilized in a crystallographic structure via suitable changes in sequence space.

### Conformational plasticity on ligand binding

**Figure 3:**
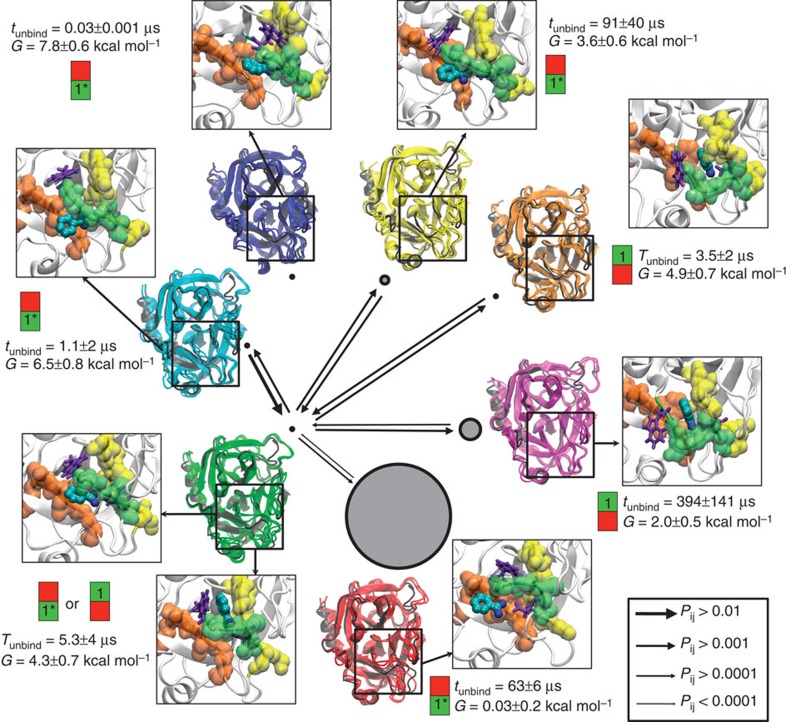
Benzamidine binding to different Trypsin conformations. Trypsin conformations with Benzamidine-bound and the binding mode of Benzamidine. The seven conformational states shown are equal to the six apo states shown in Fig. 1, plus the yellow conformation that is only found with Benzamidine-bound. The binding pocket conformation is defined by three loops: the yellow loop (residues 187–194) with Asp189, the green loop (residues 215–221) with Trp215 and the orange loop (residues 225–230). The circles have an area proportional to the equilibrium probability of the respective conformation, given that Benzamidine is bound, *π*_*i*_. Their respective relative free energies *G*=−*k*_B_*T* ln *π*_*i*_ and the unbinding times *t*_unbind_ (mean first passage time to unbinding) are given. The arrows indicate the transition probabilities for direct transitions between the different states. The binding mode (pocket 1 or 1*) is indicated by the green square with ‘1' or ‘1*'.

The overall binding free energy Δ*G*_binding_ can be calculated from the model on the basis of the stationary distribution *π*_*i*_ obtained from the MSM transition matrix and a volume correction term that accounts for standard conditions (see Methods)^23^. We obtain an overall binding free energy of 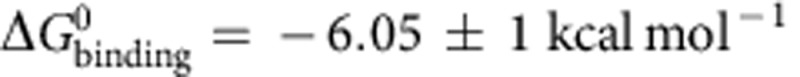 in agreement with the experimental measurement of −6.2 kcal mol^−1^ (ref. 51) and recent computational studies^23^,^52^. Figure 3 shows a representative bound conformation for each metastable protein structure.

Figure 3 shows Trypsin conformations with Benzamidine-bound and the different Benzamidine-binding modes. The conformation of the binding site is determined by three loops: the loop containing Asp189 (yellow), the loop containing Trp215 (green) and residues 225–230 (orange). Benzamidine binds either to the well-known S1-binding pocket (magenta, orange and part of the green conformations), and in the presently characterized S1* pocket (other conformations), where binding occurs ‘underneath' the Trp215 loop. Both pockets give access to Asp189, with which Benzamidine can form two stable hydrogen bonds. Consequently, both binding pockets are mutually exclusive and cannot bind two Benzamidines simultaneously. In addition to the hydrogen bonds with Asp189, Benzamidine can also form direct hydrogen bonds with Ser190 as well as indirect interactions via water molecules, confirming findings with previous metadynamics simulations^53^. However, in the present model we do not find Ser190 hydrogen bonding to give rise to the clearly separated metastable state. On the timescales of conformational changes, changes in the hydrogen bonding network are relatively fast. The ability of Benzamidine to form hydrogen bonds with Asp189 is modulated by the conformational switch Sw that varies the accessibility of Asp189 between different metastable states.

In the blue conformations Asp189 is difficult to access. In the green conformation Asp189 can be accessed via both binding pockets, but in both conformations the loop structures disturb the binding. Four conformations exhibit high binding affinities: in the magenta conformation Benzamidine binds into the widely open S1 pocket. This binding mode is most similar to the crystal structure 3PTB and has the highest conditional binding affinity, although it is not the overall most stable bound state. In the orange conformation both binding pockets are accessible but only binding into the S1 pocket occurs with high affinity. In the yellow and red conformations the S1* pocket is open and has a high conditional binding affinity. The overall stability of the bound states is determined by both, the stability of the protein structure itself and the ligand-binding affinity. In the apo form, protein structures with the S1 pocket closed are more stable; however, the highest conditional binding affinity is found for the open S1 pocket (for example, magenta X-ray structure). Therefore, there is a trade-off between favourable protein structures and high binding affinities. Overall, this trade-off is best met by the red structure where the S1 pocket is closed and the S1* pocket has a high binding affinity because of the fact that the Sw loop containing Asp189 is moved inwards (see Fig. 1b). As a result, the red bound conformation is the most stable state found in the Trypsin–Benzamidine complex.

By comparing the relative populations in the apo form (Fig. 1a) with the populations in the bound form (Fig. 3), it is apparent that the presence of the ligand significantly shifts the populations of the protein. In addition to the conformational plasticity that can be achieved by changing sequence space (see above), Trypsin also has conformational plasticity on addition of the Benzamidine ligand. Most likely, other ligands will achieve quantitatively different population changes.

### Binding kinetics and conformational kinetics

**Figure 4:**
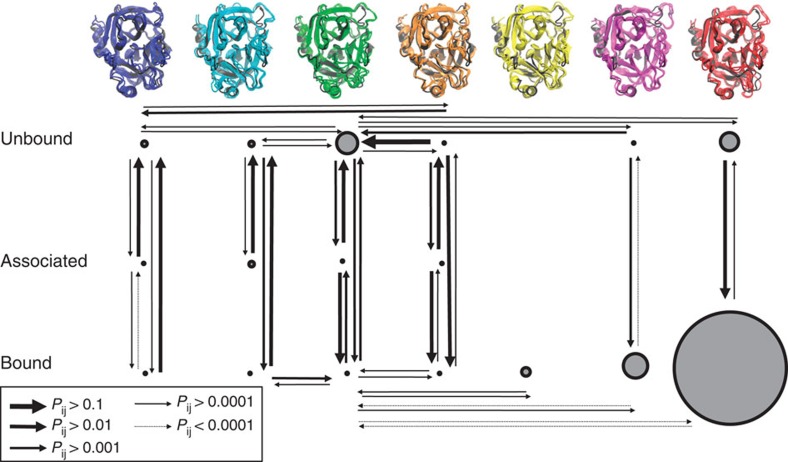
Kinetic network of binding and conformational dynamics. Kinetic network of Trypsin–Benzamidine binding and Trypsin conformational dynamics. Size of circles indicates the free energy of the states (proportional to −ln *π*_*i*_). Widths of arrows indicate transition probabilities (proportional to −ln *p*_*ij*_, see legend). The colours are identical to Fig. 1.

Figure 4 summarizes the absolute equilibrium probabilities in the simulation set-up and the kinetic network connecting bound, unbound and associated states. Both the unbound states and the bound states are only weakly connected among themselves (mainly through the green hub structure). The red structure (both bound and unbound) is kinetically most separated from the other structures. The strongest connections are between bound and unbound states and—where they exist—also associated states. Thus, the binding kinetics is dominated by kinetically separated binding channels along individual Trypsin conformations. In the blue, green and orange conformations, the ligand will bind and unbind rapidly, typically several times before a conformational change in the protein occurs. The yellow bound state is exceptional in that it is only accessible through a conformational change from the bound green state. Here the presence of the ligand induces a conformational change from the green to the yellow state. Both the magenta and the red binding channels are very stable and their bound states will only dissociate after very long waiting times.

Association (*k*_on_) and dissociation rate (*k*_off_) constants were calculated on the basis of the mean first passage times and compared with previous work and experimental data from ref. 54. The computed association rate *k*_on_=6.4±1.6 × 10^7 ^mol^−1 ^s^−1^ compares well with the experimental value 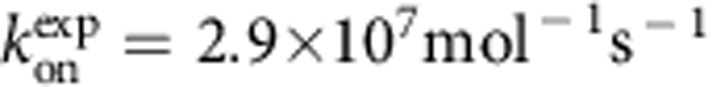, while the computed dissociation rate *k*_off_=131±109 × 10^2 ^s^−1^ is too fast compared with the experimental values and 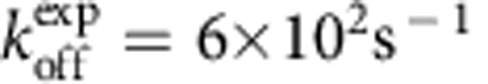, as in previous computational studies^23^. However, the uncertainty in the dissociation rate is very large as the dissociation events are poorly sampled. Methods such as TRAM^55^ could be employed in the future to substantially reduce the uncertainty of dissociation rates when the association step can be sampled well. Systematic differences in 
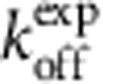
 or likely due to force field inaccuracies.

As indicated in Fig. 1, all six apo states are able to bind the ligand and possess binding times between 0.2 and 3.7 μs, with most of them ∼1 μs within our simulation set-up that corresponds to a ligand concentration of 3.7 mM.

Figure 3 reports the dissociation times conditioned on each conformation of Trypsin. The ‘unstable' binders (blue, green) have similar binding and unbinding times—here both binding and unbinding are possible on timescales of a few microseconds. The orange state is also a relatively weak binder with a dissociation time of ∼5 μs. The yellow and red states are very stable binders and have dissociation times of 60 and 90 μs, respectively. The X-ray-like (magenta) conformation is interestingly the kinetically most stable binding conformation, having an unbinding time of almost 400 μs, despite the fact that it is 2 kcal mol^−1^ less stable than the red state.

### Binding pathways and mechanisms

**Figure 5:**
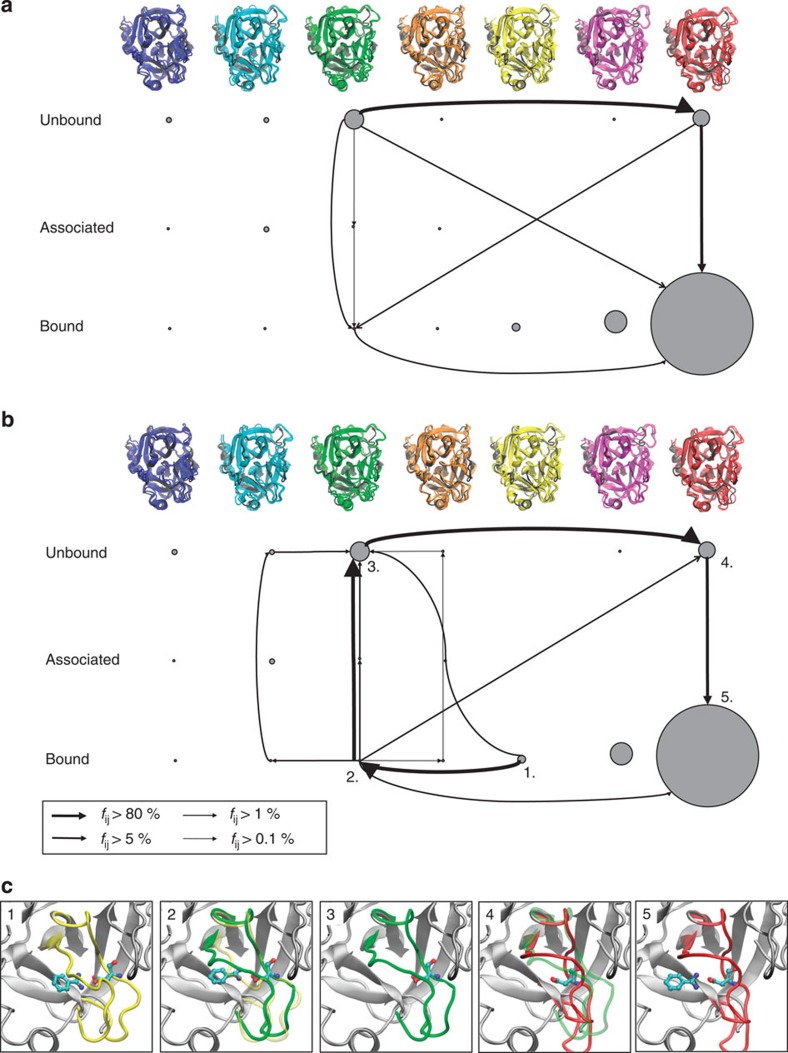
Binding and rebinding pathways. Binding and rebinding pathways with probability fluxes *f*_*ij*_ between states *i* and *j* obtained using the transition path theory. (**a**) Binding pathways from the most stable unbound (apo) to the most stable bound state. Part of the mechanism is a transition from a binding-incompetent to a binding-competent structure that includes a rearrangement of biding pocket 2 shown by the green and red loops in **c**. (**b**) Rebinding pathways from a misbound structure to the most probable bound structure. The complex is most likely to first dissociate and then reassociate, indicating that conformational selection dominates the kinetics. (**c**) Structures of the main rebinding pathways in **b**.

The transition path theory^56^ in the MSM formulation^14^ was used to analyse the binding pathways and rebinding pathways, illustrating typical mechanisms of protein–ligand binding. Figure 5a shows the predominant set of binding pathways to the most stable apo conformation (green) ending up in the most stable complex conformation (red). Since the green conformation is a state with a small conditional binding affinity, there is a conformational switch towards the red structure during the binding that can either occur in the unbound ensemble or after ligand association. Here the transition in the unbound ensemble is slightly preferred to the transition in the complex state. As a result of this conformational switch, the associated pathway is slow and occurs at timescales of the conformational changes. This binding mechanism is dominated by the conformational selection pathway^43^.

Figure 5b shows a rebinding pathway in which the kinetically very stable yellow bound conformation unbinds and rebinds to the most stable bound conformation (red). The fact that rebinding occurs predominantly through the unbound state and not directly is another indicator that conformational selection and kinetic partitioning play an important role in the present protein–ligand pair. The structures corresponding to the main rebinding pathway are shown in Fig. 5c.

On the other hand, the population changes between unbound and bound conformations are a hallmark of induced fit-type binding^43^. A more direct evidence for induced fit is the existence of the yellow conformation that is only observed in the bound state. In this state, the opening of pocket 2 is induced by Benzamidine. Therefore, Trypsin–Benzamidine exhibits features of both induced fit and conformational selection.

## Discussion

By combining extensive MD simulations and Markov models, we have explored the conformational dynamics of Trypsin and its coupling to the inhibitor binding and dissociation of Benzamidine. A key to sample the rare-event transitions was to break down conformational and binding-unbinding events into smaller transitions between Markov model microstates. By conducting a Markov model analysis during the simulation procedure and restarting trajectories in the newly found states that were yet under-sampled, we could obtain data to parametrize a Markov model on timescales that are much beyond those that can be explored by straightforward MD simulation. While this procedure was mostly carried out manually in this work, future studies may be able to find fully automated ways of adaptive simulation^52^,^57^,^58^.

We have found six distinct conformations in the apo state of Trypsin that interconvert slowly, on the order of tens of microseconds. These transitions are governed by conformational switches that control the accessibility of two binding pockets, thus affecting both affinity and kinetics of binding.

The binding kinetics exhibits features of both the conformational selection and induced fit binding models. The main binding pathways occur by first selecting a binding-competent (high affinity) conformation and then binding. However, on binding the populations are shifted and the existence of at least one new protein conformation is induced by the ligand, which is a hallmark of induced fit. In situations of high ligand concentrations, the binding rate is limited by the transition rates between apo conformations. At lower concentrations, protein–ligand encounter complexes are rare, the conformational kinetics in the apo state have averaged out and the protein state is picked from equilibrium. Nevertheless, even in this situation, the conformational kinetics and binding kinetics are intertwined as the different conformations possess different conditional binding affinities, that is the relative stabilities of conformations change on binding. Therefore, binding events mix with slow conformational transitions and can be affected by escape times from conformations that live tens or even hundreds of microseconds before relaxing to the most stable bound state.

Overall, the binding/unbinding kinetics of Trypsin–Benzamidine cannot be faithfully described as a two-state process because the slowest transitions are not due to binding/unbinding. The Markov model parametrized here could be used as a suitable effective model that could, for example, be embedded into a particle-based reaction-diffusion simulation^59^ to simulate the effects of protein and inhibitor distribution in a cell or an assay.

Besides Asp189, which is known to be critical as a recognition site for the Benzamidine inhibitor, a number of protein residues have been identified in this study to critically affect binding. These include the following: Trp215 that acts as a ‘lid' that can open and close the binding pocket as well as Val217, Gln192 and Gly219 that stabilize the lid in the closed state. This is corroborated by the fact that for various other Trypsin-like serine proteases, a similar role of Trp215 has been found. These findings suggest mutational studies that could be carried out in an experimental essay to test our simulation predictions.

Perhaps the most remarkable insight of this study is that the various Trypsin conformations can be accessed and stabilized in different ways. For five of the six states corresponding X-ray structures in the pdb data bank could be identified that exhibit the same structural features at the binding site. For the sixth state structures with similar features but no direct correspondence at the binding site were found. Thus, our wild-type simulations explore a space of conformations that can be individually stabilized by adding ligands or making suitable changes in protein sequence. Thus, Trypsin has a large degree of conformational plasticity as it was, for example, reported for kinases^60^. This observation might hint towards a general principle of conformational plasticity in protein receptor-binding sites that deserves further experimental and theoretical studies.

## Methods

### MD simulation set-up

The MD set-up and parameters for Trypsin and Benzamidine are identical to the settings used in ref. 23. The set-up was based on the X-ray structure of a Trypsin–Benzamidine complex (PDB 3PTB). Trypsin was modelled using the AMBER 99SB force field^61^ and the ligand with the general AMBER force field^62^. The system was solvated with TIP3P water molecules^63^ and neutralized with chloride ions. The simulation contained one calcium ion as found in crystal structure 3PTB coordinated by residues Glu70, Glu77 and Glu80. The data set used for the following analyses contained the 491 trajectories of 100 ns used in ref. 23. Additional MD simulations were carried out using the ACEMD programme^64^ on a in-house GPU cluster. Four trajectories of 1 μs and forty-eight trajectories of 2 μs were started from different starting structures (unbound and bound, different metastable states) in multiple rounds of manual selection. The total simulation time sums up to 149.1 μs. Full configurations were written every 100 ps for analysis.

### MSM and validation

The MSM was obtained from the MD simulation data by combining functionalities of the programme EMMA^65^ ( http://pyemma.org). Initially, various definitions of microstates were tested. We found that the distances between the Trypsin residues were required to resolve the conformational changes of the protein. Using minimum distances between all 234 residues would result in 27,261 protein distances, which is numerically unfeasible; therefore, distances between groups of two subsequent residues were used as input coordinates. The slow linear subspace of these input coordinates was then estimated by computing a time-lagged independent component analysis (TICA)^18^,^66^, and a dimension reduction was achieved by projecting on the five slowest TICA components. Uniform-distance clustering^67^ was employed to obtain an initial set of 139 microstates. An analysis of the microstates with various numbers of clusters showed that, while the microstates clearly distinguished protein conformations, they did not clearly distinguish between unbound and bound states. Hence, each microstate was further split into bound state and all remaining states, based on the Asp189-Benzamidine distance with a cutoff of 6 Å. This yielded a new set of 237 microstates. The cutoff was determined on the basis of the histogram of the Asp189-Benzamidine distance of all trajectories (see Supplementary Fig. 2). The coordinates of microstates in the first three TICA coordinates is shown in Supplementary Fig. 8.

The transition matrix 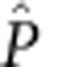 was calculated using the maximum likelihood reversible transition matrix using the quadratic optimizer described in ref. 67, that is,


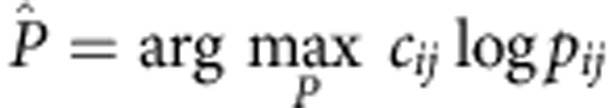



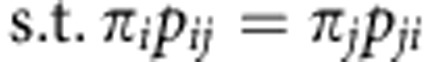


where *π*_*i*_ is the stationary probability of microstate *i*.

The Markov model was validated in two ways. First, we computed the implied timescales^46^, that is, the Markov model's relaxation timescales as a function of the lag time *τ*. Moving block bootstrapping^68^ with block size *τ* was employed to compute statistical uncertainties. Supplementary Fig. 3 shows the expectation value and the 1*σ* confidence interval of *τ*-dependent timescales obtained like this. Timescales become constant within statistical error at a lag time of ∼20–30 ns. Here *τ*=30 ns was chosen to estimate the final Markov model. As a second validation step we conducted a Chapman–Kolmogorow test of the Markov model as described in ref. 67. The Markov model estimated at 30 ns is consistent with simulation data at all lag times up to 1 μs (Supplementary Fig. 3).

### Metastable states and coarse-graining

The PCCA++ method^69^ implemented in pyEMMA was used to compute the metastable sets of microstates. Given that a gap is found after the sixth relaxation timescales, seven metastable sets were identified. As in our simulation set-up the binding process is fast compared with the conformational changes, these sets mostly lumped bound and unbound states. Therefore, these seven sets were further divided into up to three subsets each by separating bound, unbound and associated sets (see above). The resulting sets are found in Figs 1, 3 and 4, and using different colours for different metastable conformations.

For each metastable set of microstates, the binding free energy of that metastable set is computed by comparing the probabilities of its bound microstates and its unbound microstates:


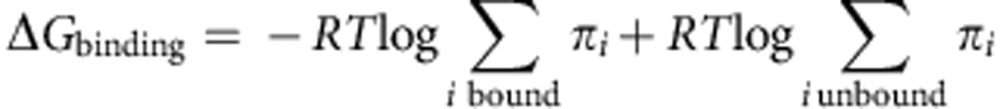


To compare to experiment this number needs to be corrected by the reference volume of the experiment compared with the simulation box, which corresponds to ∼3.1 kcal mol^−1^ in our case^23^.

Figures 1, 2, 3, 4 also show coarse-grained transition probabilities as arrows. These probabilities were obtained by coarse-graining of microstate transition probabilities (*i*,*j*) to macrostate transition probabilities (*I*,*J*) with:


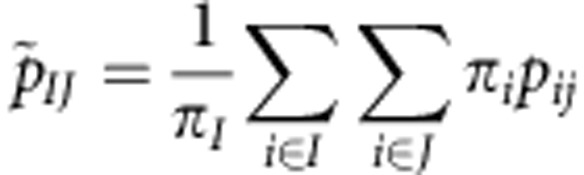


where 
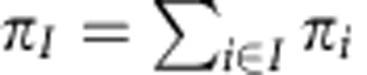
 is the macrostate equilibrium probability. Note that this coarse-graining approach only provides a qualitative illustration of kinetics and connectivity. To obtain a transferable coarse-grained model, different coarse-graining methods^70^,^71^ should be used.

The mean first passage times are computed as described in ref. 72 using pyEMMA. To obtain a coarse-grained mean first passage time from set *I* to set *J*, set *J* is defined as a target set, and the mean first passage time is then obtained as a usual expectation value:


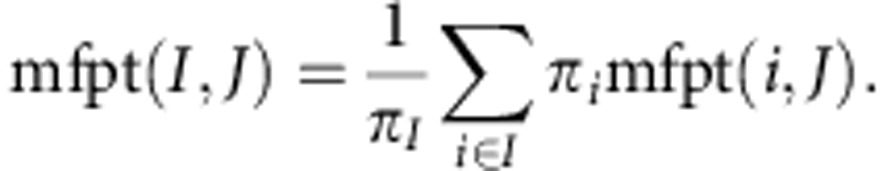


### Transition path theory

To compute the transition path fluxes shown in Fig. 5, transition path theory^56^,^73^ was employed using the expressions for transition matrices derived in ref. 14. Because the Markov model employed here is reversible, the transition path flux can be computed from the forward committor probabilities 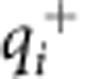 as:


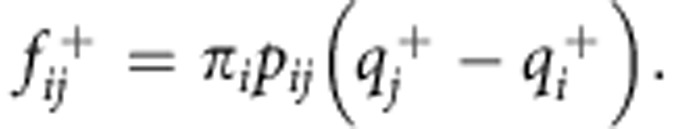


## Additional information

**How to cite this article:** Plattner, N. & Noe, F. Protein conformational plasticity and complex ligand-binding kinetics explored by atomistic simulations and Markov models. *Nat. Commun.* 6:7653 doi: 10.1038/ncomms8653 (2015).

## Supplementary Material

Supplementary Information Supplementary Figures 1-8 and Supplementary Reference
