# Supplementary material for: Protein conformational plasticity and complex ligand-binding kinetics explored by atomistic simulations and Markov models
Source: Nat Commun. 2015 Jul 2;6:7653. doi: 10.1038/ncomms8653 (PMC4506540; doi:10.1038/ncomms8653)
Supplement: Supplementary Information — Supplementary Figures 1-8 and Supplementary Reference [file ncomms8653-s1.pdf]

Supplementary information: **Protein conformational plasticity and complex ligand binding kinetics explored by atomistic simulations and Markov models**

Nuria Plattner* and Frank Noé†

Department of Mathematics, Computer Science and Bioinformatics, FU Berlin, Arnimallee 6, 14195 Berlin, Germany

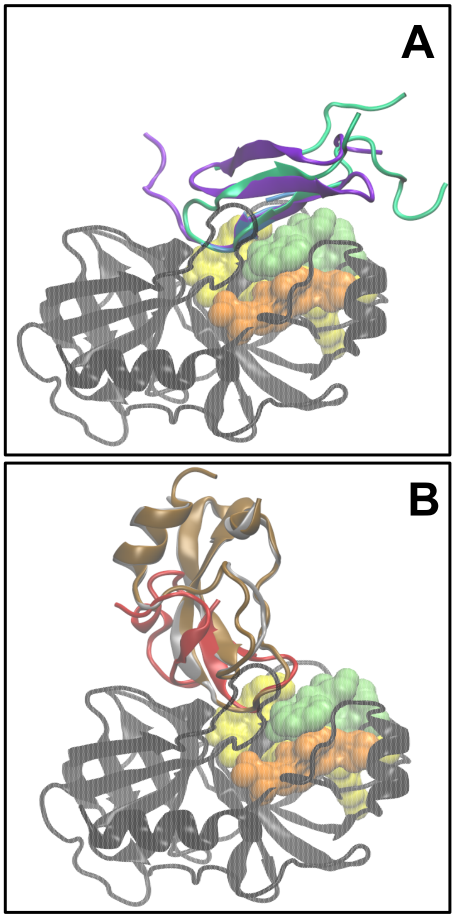

Supplementary figure 1: **Effects of conformational changes on substrate binding**
Trypsin (PDB 3PTB, dark grey) is shown with the main loops forming the benzamidine binding pocket: residues 187-194 (yellow), residues 215-221 (green) and residues 225-230 (orange). Complexes with different substrates or substrate-mimicking inhibitors are superimposed to Trypsin. A) Schistocerca Gregaria protease inhibitor (PDB 2XTT, violet), Bowman-Birk inhibitor (PDB 1TAB, green), Vasopressin (PDB 1YF4, blue). B) Bovine pancreatic Trypsin inhibitor (PDB 3DTK, brown), Alzheimer's amyloid β-protein precursor (PDB 1TAW, grey), Spinacia Oleracea Trypsin inhibitor (PDB 4AOQ, red).

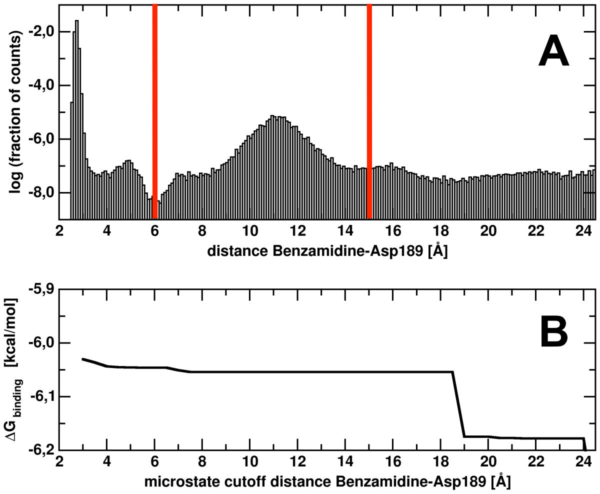

Supplementary figure 2: **Definition of bound, associated and unbound states**
A) Histogram of the Asp189-Benzamidine distance computed from all trajectories. The first maximum at about 3 Å originates from the most stable bound state the second maximum at about 11 Å originates from the associated (pre-bound) states. Based on this histogram, cutoffs of 6 and 15 Å (red lines) were used to distinguish bound, associated and unbound states. B) Binding free energy calculated based on the stationary distribution as a function of the cutoff. For each cutoff the binding free energy is determined as the free energy difference between states inside and outside the cutoff.

*E-mail: nuria.plattner@fu-berlin.de
†E-mail: frank.noe@fu-berlin.de

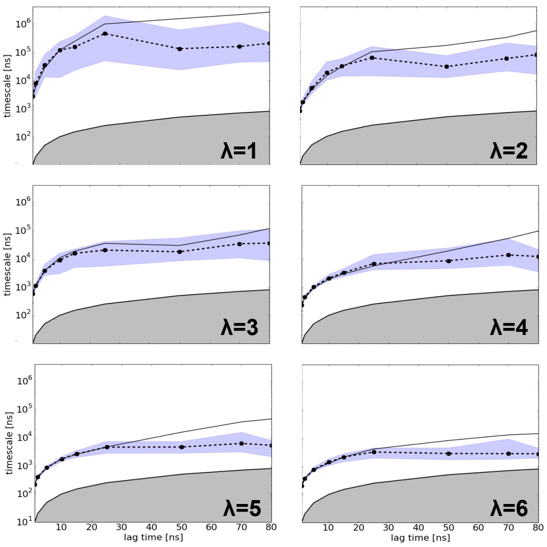

Supplementary figure 3: **Implied timescales**
Implied relaxation timescales for the eigenvalues $\lambda = 1$ to $\lambda = 6$ calculated from the transition matrix at different lagtimes. Average implied timescales (dotted lines) and confidence intervals of 95 % (colored areas) have been calculated by bootstrapping and are compared to the implied scale from the maximum likelihood transition matrix (solid lines). The grey areas mark the values $t_i < \tau$, where the lag time exceeds the time scale in order to highlight regions where timescale estimates would be unreliable as the estimated processes would already have decayed there.

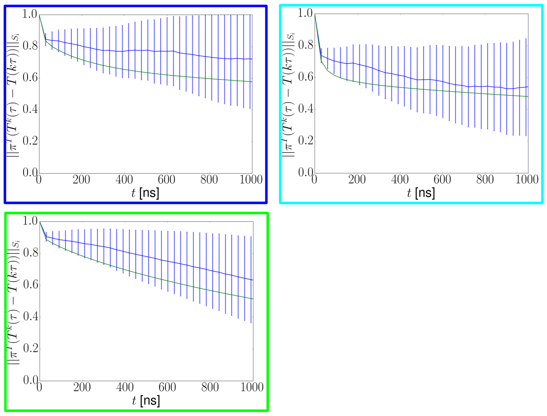

(a)

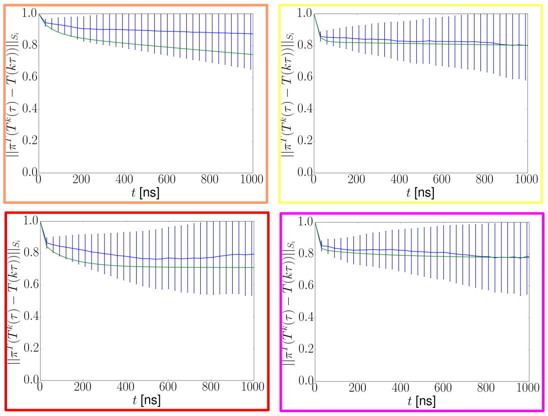

(b)

Supplementary figure 4: **Chapman-Kolmogorov tests**
(a) Chapman-Kolmogorov tests as described in [1] for predominantly unbound metastable states (a) and predominantly bound metastable states (b). The frame color encodes the protein conformation (see main text Fig. 1). Blue line and error bars: probability remaining in the given metastable state after starting all probability in that state, observed from trajectory data after time t. Green line: prediction of the Markov model transition matrix $\mathbf{P}(\tau)$ estimated at $\tau = 30$ ns.

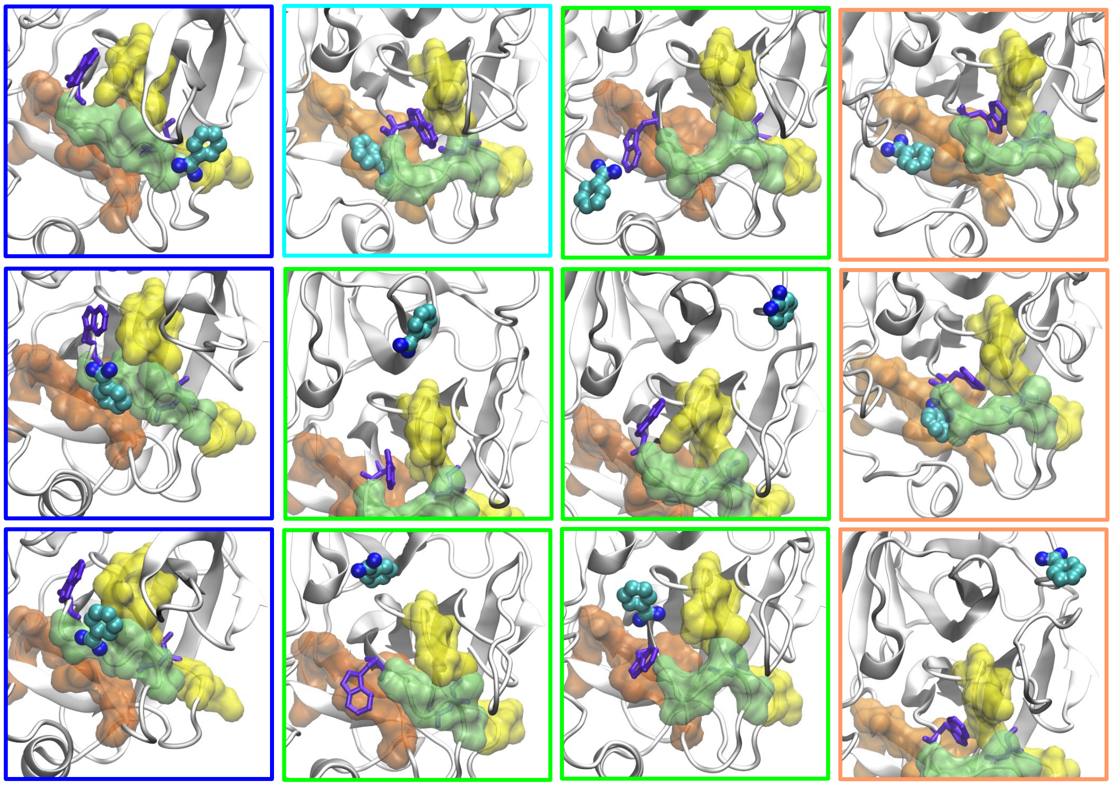

Supplementary figure 5: **Structures of associated states**
Representative structures for associated states in different macro-states. The color coding of the picture borders corresponds to the color coding of the conformations in Fig. 1 (main text). The conformations shown are relatively short-lived and thus not always separated into different metastable conformations. Thus, different associated state structures are found in the same metastable conformation.

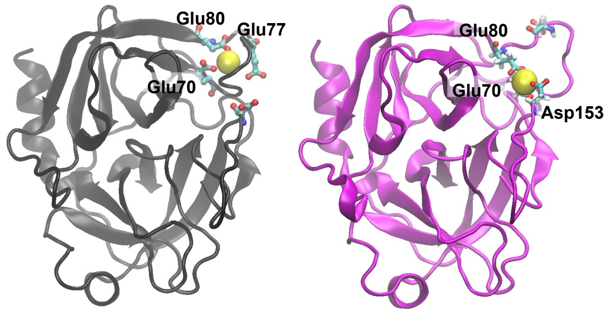

Supplementary figure 6: **Calcium coordination**
Change of the calcium coordination between the PDB structure 3PTB (left) and the magenta conformation (right). In all other conformations the calcium binding is the same as in the PDB 3PTB.

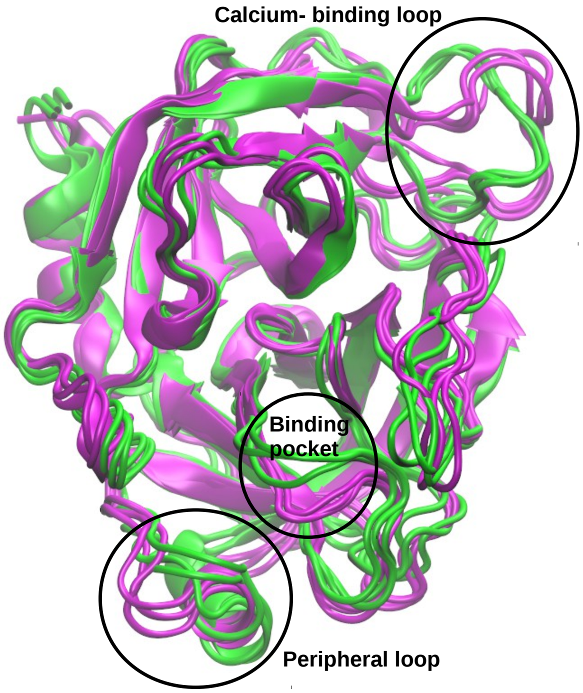

Supplementary figure 7: **Differences between magenta (X-ray like) and green (hub) conformations**
Differences between the green kinetic hub conformation and the magenta X-ray like conformations are highlighted here, including the calcium binding loop (residues 71 to 79), a binding pocket forming loop (residues 214 to 220) and the peripheral loop+helix formed (residues 164 to 177).

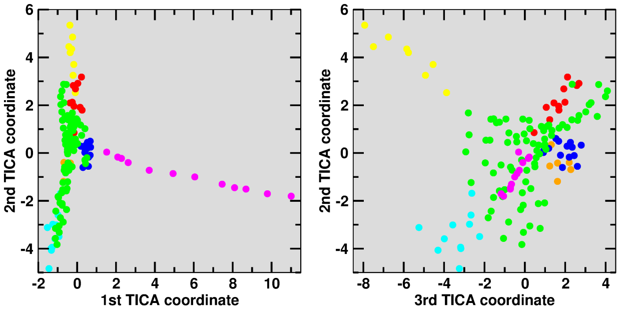

Supplementary figure 8: **TICA coordinates and PCCA sets**
Coordinates of microstates (bullets) and PCCA sets plotted in the space of the 1st/2nd (left) and 2nd/3rd (right) TICA coordinate. The colors correspond to the color coding introduced in Figure 3 (main text).

References

[1] Prinz, J.-H. *et al.* Markov models of molecular kinetics: Generation and validation. *J. Chem. Phys.* **134**, 174105 (2011).
